# Effects of Caffeine Intake Combined with Self-Selected Music During Warm-Up on Anaerobic Performance: A Randomized, Double-Blind, Crossover Study

**DOI:** 10.3390/nu17020351

**Published:** 2025-01-19

**Authors:** Bopeng Qiu, Ziyu Wang, Yinkai Zhang, Yusong Cui, Penglin Diao, Kaiji Liu, Juan Del Coso, Chang Liu

**Affiliations:** 1 School of Strength and Conditioning Training, Beijing Sport University, Beijing 100084, China; qiubopeng@bsu.edu.cn; 2 China Swimming College, Beijing Sport University, Beijing 100084, China; 2021210263@bsu.edu.cn; 3 China Wushu School, Beijing Sport University, Beijing 100084, China; 2020210096@bsu.edu.cn; 4 Sports Coaching College, Beijing Sport University, Beijing 100084, China; 5 School of Sport Science, Beijing Sport University, Beijing 100084, China; 6 Sport Sciences Research Centre, Rey Juan Carlos University, 28943 Fuenlabrada, Spain

**Keywords:** ergogenic aid, music, caffeine supplementation, exercise performance

## Abstract

Background: Both listening to music during warm-up and consuming caffeine before exercise have been independently shown to enhance athletic performance. However, the potential synergistic effects of combining these strategies remain largely unexplored. To date, only two studies have reported additional benefits to combining music during warm-up with a caffeine dose of 3 mg/kg on taekwondo-specific performance tasks. However, these studies did not evaluate whether this combination produces additive or synergistic effects on other types of sports performance. The present study aimed to assess the effects of listening to music alone or combined with caffeine intake on performance in the Wingate anaerobic test (WAnT) in physically active subjects. Methods: Twenty-four physically active male participants took part in this randomized, double-blind, crossover experiment. Participants underwent WAnT performance evaluations under three conditions: (i) no intervention (control; CON); (ii) music plus placebo (Mus + PLA), involving the intake of a placebo (maltodextrin) 60 min prior and self-selected high-tempo music during warm-up; and (iii) music plus caffeine (Mus + CAF), involving the intake of 3 mg/kg of caffeine 60 min prior and self-selected high-tempo music during warm-up. Under all conditions, participants wore the same Bluetooth headphones (with or without music), performed a 10 min standardized warm-up, and completed the 30 s WAnT with a load of 7.5% of their body weight on a calibrated ergometer. Power output was recorded at a frequency of 1 Hz throughout the exercise. The Feeling Scale was assessed both before and after the exercise test, while heart rate (HR) and the rating of perceived exertion (RPE) were measured immediately following the exercise. Results: Mus + PLA and Mus + CAF significantly improved peak power, mean power, and total work compared with CON (*p* < 0.05). Furthermore, peak power was higher in Mus + CAF than in Mus + PLA (*p* = 0.01). Post-exercise HR and RPE showed no significant differences across conditions (*p* > 0.05). Regarding the Feeling Scale (FS) before exercise, the Mus + PLA and Mus + CAF conditions showed significantly higher scores than CON (*p* < 0.05), while no differences were found after exercise. The perceived fitness metrics displayed no significant differences among conditions (*p* > 0.05), except for self-perceived power, which was higher in Mus + CAF than in CON (*p* = 0.03). Conclusions: Self-selected music during warm-up, either alone or combined with caffeine, significantly enhanced several WAnT performance metrics, including peak power, mean power, and total work. Remarkably, combining music with caffeine further improved peak power and increased self-perceived power compared with music alone. While listening to self-selected music during warm-up provided measurable benefits on anaerobic exercise performance, the combination of music and caffeine demonstrated additive effects, making it the optimal strategy for maximizing anaerobic performance.

## 1. Introduction

Anaerobic capacity is a critical component of athletic performance, contributing not only to short bursts of high-intensity exercise [1] but also to the sprint phases during endurance competitions [2]. In recent years, sports science practitioners have proposed numerous strategies for improving anaerobic performance, including those related to psychological interventions [3,4], nutritional supplementation [5,6], and physical training, with a particular focus on warm-up techniques aimed at enhancing performance [7,8]. Despite differing mechanisms of action, these strategies share the common goal of enhancing anaerobic performance when implemented shortly before the onset of exercise.

The targeted manipulation of psychological factors, such as motivation, focus, and arousal levels, in the minutes leading up to exercise can significantly enhance sports performance [9]. In this context, the use of music before or during exercise as a tool for emotional expression and psychological conditioning has garnered significant attention and research interest [10]. Specifically, the potential benefits of listening to music in an exercise context have been associated with improved mood, regulation of arousal, enhanced motivation, reduced perception of exertion, and increased physiological efficiency [11,12,13,14]. A recent meta-analysis encompassing 139 studies highlighted that music significantly enhances exercise performance [10]. Additionally, a separate review demonstrated that music can improve handgrip strength and performance in the Wingate anaerobic test (WAnT) [15]. The enhancement of exercise performance through music is notably influenced by various moderating factors, including the music’s intensity, tempo, and type, the timing of its use (before or during exercise), and an individual’s personal music preferences [16,17,18,19]. Two studies by Ballmann et al. found that muscular anaerobic performance and motivation for exercise could be improved under preferred music conditions [20,21]. However, further research is needed to identify the optimal scenarios for maximizing anaerobic performance with music before exercise. Given that listening to music during competitions is typically prohibited for clear safety and performance reasons [22], using pre-exercise music during the preparation period may offer a more practical and effective strategy for enhancing anaerobic performance.

Beyond psychological strategies like music, physiological interventions can be equally critical to optimizing performance. In recent years, sports nutrition has garnered significant attention as a critical factor influencing athletic performance [23]. In particular, caffeine (CAF) has been widely used in athletic competition [24,25] due to its status as one of the most popular psychoactive substances in the world [26]. Many studies have investigated the ergogenic effects of caffeine in various exercise situations [27,28,29]. To be specific, caffeine enhances performance by blocking adenosine receptors, which reduces fatigue perception and stimulates the central nervous system. This stimulation triggers the release of neurotransmitters such as catecholamines, dopamine, and acetylcholine, creating a combined effect that delays fatigue and boosts performance [30,31,32,33]. Additionally, caffeine may enhance sarcoplasmic reticulum calcium release, increase sodium–potassium pump activity, and improve excitation–contraction coupling, which collectively contributes to increased motor unit recruitment [34]. However, some of these latter effects are observed only with very high doses of caffeine, often in the range of supraphysiological levels. The broad range of caffeine’s mechanistic actions makes it ergogenic for both aerobic and anaerobic exercise performance, regardless of exercise duration [35]. Additionally, a meta-analysis by Grgic et al. [36] found that caffeine intake significantly increased the mean power output (+3%) and peak power output (+4%) in the lower-body WAnT.

Numerous meta-analyses have confirmed that anaerobic performance can be improved through either caffeine supplementation or listening to music independently [14,36], but only two studies have suggested that further potentiation of exercise performance can be obtained by a caffeine and music combination [37,38]. Delleli et al. [37] recently investigated the isolated and combined effects of listening to music and caffeine intake on taekwondo-specific performance tasks. Their study revealed that combining music during warm-up with a caffeine dose of 3 mg/kg significantly enhanced taekwondo-specific agility, performance in the 10 s Frequency Speed Kick Test (FSKT), and overall FSKT-specific abilities in taekwondo athletes, outperforming the effects of music and caffeine alone. Furthermore, this music + caffeine approach also produced more favorable psychological responses, including higher scores on sensory scales and lower ratings of perceived exertion (RPEs) [37]. Another study examined the effects of warm-up music combined with a low dose (3 mg/kg) of caffeine on female taekwondo athletes’ performance and psychophysiological responses during simulated combat. These findings suggest that combining caffeine with warm-up music enhances both physical performance and psychological state more effectively than either strategy alone [38]. However, both insightful studies are the only ones of their kind, and they did not determine whether the combination of caffeine and music produces an additive or synergistic effect on other types of anaerobic exercise performance.

Thus, the purpose of this research was to evaluate how self-selected music listening during warm-up, either by itself or in conjunction with caffeine consumption, affected the WAnT performance of physically active participants. We hypothesized that a synergistic effect might be achieved by combining the self-selected music with caffeine intake, i.e., listening to music and consuming caffeine in combination would result in a better ergogenic effect on WAnT performance compared with listening to music alone.

## 2. Methods

### 2.1. Participants

A randomized, double-blind, crossover study was carried out at Beijing Sport University (BSU). Because gender may have an impact on the ergogenic effects of caffeine, only male individuals were taken into consideration [39]. Additionally, only participants who used less than 50 mg/d of caffeine per day were included in order to account for variations in caffeine tolerance for individual responses [40]. Specific inclusion criteria for subjects were as follows: (i) no neuromusculoskeletal disorders; (ii) at least 3 years of training experience in high-intensity exercise; and (iii) being in a health condition that would allow them to complete the experimental tests. Participants were excluded from the experiment if they reported (i) smoking, (ii) alcohol consumption, (iii) caffeine allergy, and (iv) the presence of diseases and abnormalities of the ear or hearing.

Of the 24 males who met the inclusion and exclusion criteria, 23 successfully completed all tests in a randomized, crossover design. The anthropometric characteristics of the subjects are shown in Table 1. All participants were informed about the objectives of the experiment with potential risks before signing a written informed consent form. The study protocol was conducted in accordance with the ethical and moral standards of the Declaration of Helsinki and was approved by the Human Research Ethics Committee of Beijing Sport University (Ethics Committee No. 2023225H), and it was registered at ISRCTN (registration number ISRCTN10555501).

### 2.2. Study Design

Over a period of around three weeks, participants attended three experimental sessions and one familiarization session. To account for any lingering effects of the treatments, there was a week between each experimental session. The familiarization session was conducted prior to the formal experiment and included taking basic physical measurements, acquainting participants with the overall experimental procedure, and providing details about the WAnT. Each subject’s bicycle seat height was recorded for use in subsequent trials. On this familiarization day, the researcher recorded each subject’s self-selected music (3–6 tracks, totaling approximately 10 min) and created a personalized playlist labeled with the subject’s name to be played during the warm-up phase of the experimental trials. Then, three experimental trials were conducted in random order, with each subject’s experimental order being the result of a random assignment generated online via https://www.random.org/. All tests were administered by trained researchers, and the same personnel supervised all testing sessions.

**Figure 1:**
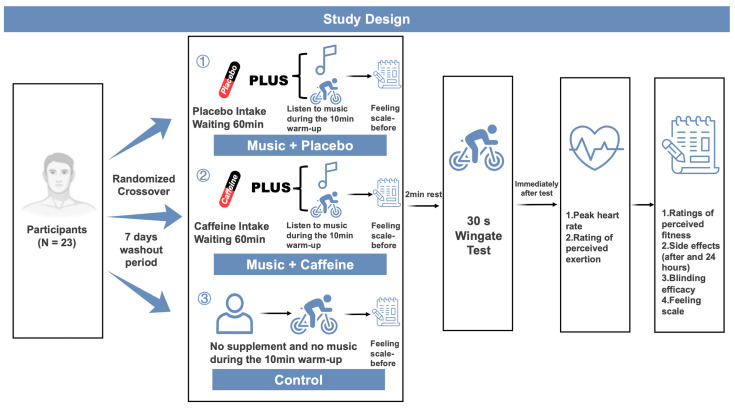
Experimental design. min = minute.

The following were the three research conditions: (i) Control group (CON): participants arrived at the laboratory and did not receive any nutritional supplementation, sat still for 60 min, and then performed a 10 min music-free warm-up on a Wingate power bike. (ii) Music + placebo (Mus + PLA): ingestion of 200 mg of placebo 60 min before exercise, followed by the same warm-up protocol as in CON but distinguished by the sequential playing of the self-selected music recorded during the familiarization session. (iii) Music + caffeine (Mus + CAF): ingestion of 3 mg/kg of caffeine 60 min before exercise followed by the same warm-up and music intervention protocol as in Mus + PLA. Under all three conditions, the warm-up was followed by the 30 s WAnT. Under all conditions, the subjects wore the same Bluetooth headphones (with or without music playing) for 10 min of warm-up. Each athlete was required to select their favorite music. The chosen music was accepted for use under the music condition as long as its tempo was ≥ 120 bpm to ensure that the music was inherently stimulating [37]. The primary outcomes of this trial were power parameters derived from the WAnT, with secondary outcomes of post-exercise heart rate (HR), RPE, Feeling Scales [41], blinding efficiency, and incidence of side effects. The experimental design is detailed in Figure 1.

### 2.3. Experimental Protocol

Ninety minutes before the commencement of the workout, the individuals in the experimental trials reported at the laboratory. For CON, no capsule supplementation or music intervention was given, and participants sat still and rested for 60 min. As in CON, participants in Music + PLA and Music + CAF received a pill containing either 200 mg of a placebo or 3 mg/kg of caffeine. They were then instructed to sit still and relax for 60 min. The capsules were identical in appearance, and ingestion was accompanied by 100 mL of water. The exercise was initiated 60 min after capsule intake to allow near-to-peak blood caffeine concentration during the WAnT [42]. The placebo capsule contained 200 mg of maltodextrin (My Protein, Manchester, UK). In the caffeine capsule, anhydrous caffeine powder was used (Nutricost, Vineyard, UT, USA), and the ISSN position on caffeine led to the selection of 3 mg/kg of caffeine [35].

Participants were given a standardized warm-up regimen following the 60 min rest period. Participants pedaled against 20% × 0.075 kg/body weight load for 10 min of warm-up cycling with or without musical intervention, with a controlled speed of 75 r/min. Then, they performed the WAnT on a Monark power cycle ergometer (Ergomedic 894E; Vansbro, Sweden). For this test, the participant was loaded with 7.5% of their own body weight and asked to ride at a maximum pedaling speed for 30 s. A researcher supervised the subject’s seated position at all times during the test. During all tests, the researcher used standardized words and actions to encourage the subjects. The fatigue index (FI) was calculated according to a formula from a previous study [43]. The researchers were blinded to the condition of the subjects’ participation during the measurement and analysis of the data.

Immediately after the WAnT, the HR was measured by having a researcher place a heart rate sensor on the participant’s chest (H9; Polar Electro Oy, Kempele, Finland). Then, the researcher queried participants about the RPE by using the Borg scale (6–20) [44].

The participants completed several questionnaires at different time points. Five minutes after the test, the participants were asked to complete the first questionnaire, which used a 0–9 point scale, with 0 being the lowest quantity of the item and 9 denoting the maximum, and to rate their impressions of power, endurance, and exhaustion throughout the Wingate test [45]. The Feeling Scale [41,46] was administered both prior to and immediately following the WAnT. In addition, 24 h following exercise testing, participants answered a second questionnaire that used a yes/no scale to gauge how the intervention affected their quality of sleep, anxiety levels, and any other discomforts [47]. Lastly, the Bang Index, which was determined by looking at how participants answered the question, “What kind of supplement do you think you received today?” was used to evaluate how well the blinding technique performed. The available options were caffeine, placebo, or “I don’t know”.

Each test session was scheduled from 8 to 12 am to minimize bias from circadian rhythm disruptions linked to supplement consumption. Subjects were encouraged to maintain the same dietary and sleep habits throughout the study, and all participants were instructed to avoid any form of strenuous exercise for 48 h prior to each test session, to avoid alcohol for the duration of the study as well as the intake of any source of caffeine or additional exercise ergogenic supplements such as creatine, taurine, and other substances. Each subject was provided with a list of caffeine-containing beverages or foods prior to the start of the experiment to control caffeine intake [6].

### 2.4. Analysis of Statistics

Mean ± standard deviation (SD) are used to display all data. The dataset’s normality was assessed by using the Shapiro–Wilk test. One-way repeated measures analysis of variance (ANOVA; 1 × 3; CON, Mus + PLA, and Music + CAF) was used to compare differences in Wingate performance outcomes, post-exercise HR, and other variables with normal distribution. When a significant main intervention effect of ANOVA was detected, post hoc comparisons with Bonferroni correction were performed. Effect sizes (ESs) for pairwise comparison were calculated with Cohen’s *d* and interpreted as follows: trivial (0–0.2), small (0.2–0.5), moderate (0.5–0.8), and large (>0.8). For non-normally distributed data, the Friedman test was used, followed by a post hoc test using the Dunn’s test. The Bang Index was used to check the validity of the blinded caffeine intake procedure implementation [48]. All statistical analyses were performed by using SPSS 26.0 (SPSS, Inc., Chicago, IL, USA) and GraphPad Prism 9.0 (GraphPad, San Diego, CA, USA) software, with the significance level set to *p* ≤ 0.05.

## 3. Results

### 3.1. Wingate Anaerobic Test Performance, Heart Rate, and Ratings of Perceived Exertion

**Figure 2:**
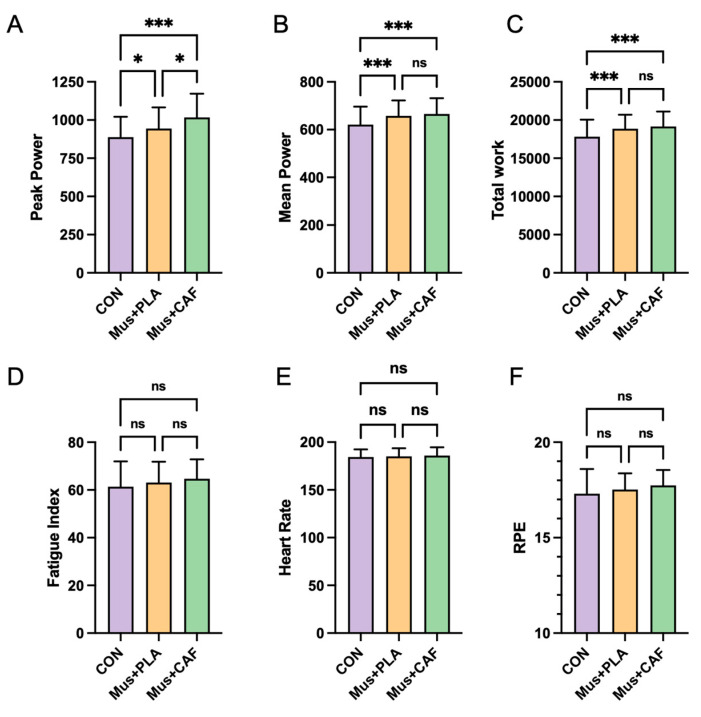
Effects of self-selected music combined with placebo (Mus + PLA) or caffeine supplementation (Mus + CAF) on Wingate anaerobic test (WAnT) performance, heart rate, and ratings of perceived exertion (RPEs) in physically active subjects compared with a control condition without music or caffeine (CON). (**A**) peak power; (**B**) mean power; (**C**) total work; (**D**) fatigue index; (**E**) heart rate; (**F**) RPE. ns = no statistical difference, * = *p* ≤ 0.05, and *** = *p* < 0.001 compared with other conditions.

WAnT performance variables for the three conditions are shown in Table 2 and Figure 2. The intervention had a significant effect on peak power, mean power, and total work (*p* < 0.05). Post hoc comparisons utilizing Bonferroni correction indicated improved peak power, mean power, and total work under the Mus + PLA and Mus + CAF conditions relative to CON (*p* < 0.05), with average improvements of 6.29% and 14.56%, respectively, for Mus + PLA and Mus + CAF in peak power. No significant differences were observed between Mus + PLA and Mus + CAF in mean power or total work (*p* > 0.05). However, the percentage improvement under the Mus + CAF condition was greater than under the Mus + PLA condition, with increases of 7.13% vs. 5.88% for mean power and 7.51% vs. 5.89% for total work. Pairwise comparisons further showed that Mus + CAF achieved significantly higher peak power than Mus + PLA (MD: 73.44 W, 95% CI: 13.77 to 133.1, *p* = 0.01, ES = 0.50). Additionally, no significant differences were observed among the three conditions in fatigue index, heart rate, or ratings of perceived exertion (Figure 2).

### 3.2. Feeling Scale

**Figure 3:**
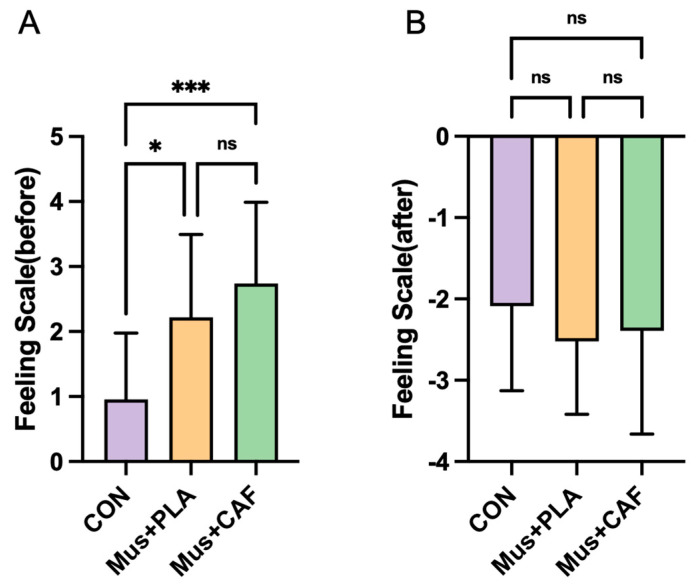
Effects of self-selected music combined with placebo (Mus + PLA) or caffeine supplementation (Mus + CAF) on the Feeling Scale measured before and after the Wingate anaerobic test (WAnT) in physically active subjects compared with a control condition without music or caffeine (CON). (**A**) Feeling Scale ratings before the WAnT; (**B**) Feeling Scale ratings after the WAnT. ns = no statistical difference, * = *p* ≤ 0.05, and *** = *p* < 0.001 compared with other conditions.

The results of the Feeling Scale for the three conditions are shown in Table 3 and Figure 3. There was a main effect of the intervention on FS–before (*p* < 0.001). Post hoc analysis revealed that FS–before scores were significantly higher under the Mus + PLA condition compared with CON (rank sum difference = −18.50, *p* = 0.02) and under the Mus + CAF condition compared with CON (rank sum difference = −28.00, *p* < 0.001). However, no significant difference was observed between the Mus + PLA and Mus + CAF conditions (rank sum difference = −9.500, *p* = 0.48). For FS–after, no significant differences were found among conditions (*p* = 0.67).

### 3.3. Perceived Fitness, Side Effects, and Blinding Efficacy

Table 4 shows the incidence of adverse effects both immediately after exercise and 24 h later, as well as subjective experiences of power, endurance, and fatigue following exercise in the three circumstances. In these respects, a significant main effect was observed for the self-reported perceived power values among the three conditions (*p* = 0.036). Post hoc comparisons revealed that participants under the Music + CAF condition reported significantly higher perceived power values compared with the CON condition (*p* = 0.025), while no statistically significant differences were observed between the other pairwise comparisons (*p* > 0.05). Additionally, no statistically significant differences among the three conditions were found in any of the other variables (*p* > 0.05). Just nine individuals under the Music + CAF condition correctly recognized that they had consumed caffeine (Bang Index = 0.09), whereas only eight participants correctly guessed that they ingested a placebo under the Music + PLA condition (Bang Index = 0.04). The observation that the right estimate was less than the ratio of chance indicates that the blinding of participants to supplement use was well carried out.

## 4. Discussion

Our findings indicate that both self-selected music combined with placebo (Mus + PLA) and self-selected music combined with caffeine (Mus + CAF) significantly enhance anaerobic performance, as evidenced by improved peak power, mean power, and total work in the Wingate anaerobic test (WAnT) compared with the control (CON) condition. Interestingly, the Mus + CAF condition demonstrated a significant improvement in peak power over the Mus + PLA condition, although no statistical differences were observed in mean power or total work between these two interventions. Caffeine or placebo intake combined with self-selected music was associated with an increased Feeling Scale compared with the CON condition. Nevertheless, subjective feelings of power were higher under the Mus + CAF condition, emphasizing again the potential interaction of caffeine and music on self-reported exercise experiences before exercise. Lastly, no significant differences in ratings of perceived exertion, heart rate, or fatigue index were observed across the three conditions, indicating that in all trials, the participants exerted at their maximum level but with better performance values in the combination of music and caffeine. Collectively, all this information suggests that the combination of caffeine intake and music before anerobic exercise may be an effective strategy to enhance performance.

Music preference and music tempo have been shown to benefit athletes to varying degrees during exercise [10,49]. Research on preferred music has shown [20] that during the first three bench press repetitions, mean speed and peak power were higher when listening to preferred music than non-preferred music. Preferred music has also been shown to result in significantly improved time trial performance, including higher relative power and shorter trial times for rowing, whereas there was no significant improvement with non-preferred music [50]. While these studies support that preferred music promotes power and athletic performance, some of the studies have also found no significant differences between preferred and non-preferred music at the level of athletic performance, which warrants further research [21,51]. One study [21] investigated the effects of listening to preferred versus non-preferred music during three sets of 15 s Wingate anaerobic tests, performed with 2 min rest intervals between sets. The study, which used music with an average tempo of 128 ± 31 bpm, found that while preferred music did not significantly enhance exercise performance compared with non-preferred music, it did increase motivation and reduce perceived exertion. It is worth noting that although the average tempo was 128 bpm, the preferred music for some subjects fell into the low- or medium-tempo category (<120 bpm). Low- or medium-tempo music appears to have a weaker effect on sports performance compared with high-tempo music (≥120 bpm) [10].

We found that the Mus + PLA condition significantly improved anaerobic performance compared with the control group, which may be attributed to increased subjective feelings of energy or vitality. The properties of music, such as high-tempo and preferred music, are thought to modulate increases in arousal with concomitant increases in performance [52,53]. Additionally, listening to music can cause changes in physiological factors [54]; for example, it has been demonstrated that music has been shown to increase adrenaline levels during exercise [55], which may ultimately affect muscle activation and metabolic responses during subsequent exercise. Meanwhile, listening to preferred music has been shown to improve pain tolerance [56]. Preferred music has a greater ability to shift attention from the discomfort of movement to external musical stimuli, which can reduce the RPE [57]. In the present study, listening to preferred high-tempo music also had a positive effect on subjective feeling, which may promote anaerobic exercise capacity, even though the mechanism of correlation has not yet been demonstrated.

We found that combining self-selected music with caffeine enhanced peak power more effectively than combining it with a placebo. In our study, there was a statistically significant difference in peak power between the Mus + CAF and Mus + PLA conditions. A meta-analysis of studies about the effect of caffeine on anaerobic cycling performance indicated that CAF increases anaerobic capacity during Wingate tests despite variations in study specifics [36]. The current study provides novel information, as it suggests for the first time that the effect of CAF on anaerobic performance can even be amplified with music listening during warm-up. The mechanisms involved may be varied; specifically, CAF acts as a powerful central nervous system stimulant [30,58], increases neuromuscular recruitment [59], and enhances muscle contraction [60]. In this context, it is possible that music interacted with caffeine’s mechanisms of action in Mus + CAF by enhancing dopamine release [61], thereby boosting motivation and readiness for exertion before exercise.

Although the values in the Feeling Scale were not statistically different between the Mus + CAF and Mus + PLA conditions before exercise (and both were different from CON; see Figure 3), the Mus + CAF condition was the condition with higher values in the Feeling Scale, reflecting enhanced subjective feelings just before the onset of the all-out exercise test. A study has shown that listening to exciting music during a warm-up increases catecholamines [54], which may ultimately influence an individual’s self-feeling, subsequent muscle activation, and metabolic responses during exercise. Additionally, due to CAF-induced dopamine neurotransmitter release, this likely led to a positive effect on an individual’s mood [62], which further adds to the increased neural recruitment of muscles and power facilitated by high-tempo music. In addition, caffeine’s effects on neurotransmitter release, including dopamine and catecholamines, interact with the neuromuscular system to enhance motor output and potentially reduce perceived exertion during exercise [63]. Similarly, high-tempo music modulates hormonal responses, including plasma epinephrine, which influences both psychological states and physical performance [55]. These mechanisms highlight the synergistic interaction between caffeine and music in positively influencing neural and hormonal pathways, enhancing readiness and motivation before exercise. The physiological, neurological, and psychological mechanisms underlying the effects of caffeine and music on muscle contraction likely involve complex interactions. For instance, caffeine’s role as a central nervous system stimulant is thought to contribute to increased motor unit recruitment and muscle contraction [27]. Similarly, music, particularly high-tempo and preferred music, has been suggested to elevate catecholamine levels, potentially enhancing arousal, which may synergize with the neuromuscular activation facilitated by caffeine [49]. However, it is important to note that these mechanisms were not directly measured in this study and remain speculative based on the prior literature. To date, only studies on taekwondo [37,38] have shown that CAF combined with high-tempo warm-up music can improve parameters and psychological states related to exercise performance. Although taekwondo differs from Wingate testing in terms of movement patterns and duration, the findings of these studies, along with the outcomes of our study, support the notion that the combination of music and caffeine provides an ergogenic effect prior to exercise.

Given that the Wingate test is highly relevant to other anaerobic-based sports events, the results presented in this study are particularly relevant to athletes involved in high-power-output activities [64]. From a practical perspective, integrating moderate caffeine intake around 1 h before exercise with high-tempo, preferred music during warm-up may serve as an effective, low-cost approach to maximize power output and motivation in short-duration, high-intensity events. Coaches and researchers should tailor pre-exercise strategies based on athletes’ individual preferences and caffeine tolerance to achieve optimal results. These findings can guide practical strategies for athletes and coaches aiming to enhance performance in anaerobic sports. Furthermore, the observed synergy between music and caffeine suggests that combining psychological and physiological interventions may yield greater benefits than either approach alone, offering new perspectives on performance enhancement through sports nutrition and psychology. Importantly, as with the previous study [43], our research found no significant side effects associated with 3 mg/kg caffeine intake. In line with these findings, a relatively small dose, such as 3 mg/kg, taken in the morning or midday, has been shown to have low incidence of side effects [45]. Nevertheless, several drawbacks of the present study warrant discussion to enhance the comprehension of the findings’ extent.

First, the psychological measures used in this study were limited. Additionally, physiological markers such as blood lactate levels were not assessed. The relationship between blood lactate concentration and the combined effects of caffeine and music is an area of interest. Caffeine has been shown to reduce perceived exertion and delay the onset of fatigue during submaximal-intensity exercise [34]. However, caffeine intake increases blood lactate concentration, a metabolic marker that reflects caffeine’s ability to enhance glycolytic pathways, following all-out exercise trials, ultimately contributing to improved performance [65]. Additionally, music’s role in enhancing focus and reducing discomfort may indirectly influence lactate clearance rates [66]. So, future investigations are needed to determine if the combination of caffeine and music produces greater blood lactate concentrations, suggesting a combined effect of both treatments on enhanced glycolytic activity. Fast-twitch muscle fibers, which are primarily involved in anaerobic efforts, may benefit from the combined effects of music and caffeine, as both stimuli promote enhanced activation and recovery. This is because fast-twitch fibers primarily rely on anaerobic glycolysis for energy production, making them more responsive to interventions that enhance glycolytic pathways. Additionally, fast-twitch fibers are more sensitive to high-frequency motor neuron firing, making them potentially more receptive to caffeine and music interventions than slow-twitch fibers. Another potential limitation of this study is the lack of a separate “caffeine-only” condition (caffeine [+] + music [−]). Without this condition, we are unable to ascertain whether music and caffeine have an additive or synergistic effect under the Mus + CAF condition. Finally, while this study used a low dose of caffeine (3 mg/kg) with no reported serious side effects, it is crucial to address the potential risks of excessive caffeine intake in athletes, especially with doses of caffeine over 6 mg/kg, as these doses increase the prevalence and magnitude of side effects [67]. The overconsumption of caffeine can lead to adverse effects such as dehydration, anxiety, and disrupted sleep patterns, which may impair recovery and overall performance [35]. Future research should focus on establishing safe and effective strategies for caffeine use in athletic populations, particularly when combined with complementary interventions like music to maximize performance benefits while minimizing risks. Identifying the minimal ergogenic dose of caffeine is crucial to delivering a performance-enhancing effect while minimizing the likelihood of adverse side effects.

In general, even if this study offers insightful information, the results should be evaluated in light of the limitations of the current experimental setup, especially in light of the absence of post-exercise physiological measures and specific settings. By assessing more physiological and psychological factors and adding additional conditions, future research might improve our knowledge of how caffeine and music affect performance.

## 5. Conclusions

This study demonstrates that listening to a list of self-selected music during warm-up may be an effective strategy to enhance acute anaerobic performance, as evidenced by increased power output and total work in the Wingate test. The addition of caffeine amplified the benefits of music by further improving peak power and subjective perceptions of power, with no reported adverse effects. These findings suggest that the integration of listening to high-tempo, self-selected music with caffeine is a promising approach to optimizing anaerobic-based performance. As a practical application, athletes and coaches of anaerobic-based sports can incorporate self-selected music during warm-up protocols combined with a moderate caffeine dose (e.g., 3 mg/kg) to enhance anaerobic performance. These strategies, when combined, may improve pre-exercise mood and perceived readiness. However, further research is needed to investigate the broader applicability of this strategy, particularly in other sports events (e.g., endurance-based exercise activities) and among female athletes.

## Figures and Tables

**Table 1:** Anthropometric characteristics of the individuals.

Variables	Mean ± SD
Age (year)	21.00 ± 1.62
Body height (cm)	181.43 ± 4.64
Body weight (kg)	76.09 ± 7.17
BMI (kg/m^2^)	23.08 ± 1.47

Note: Data are presented as means ± SDs. BMI = body mass index.

**Table 2:** Effects of self-selected music combined with placebo (Mus + PLA) or caffeine supplementation (Mus + CAF) on Wingate anaerobic test (WAnT) performance, heart rate, and ratings of perceived exertion in physically active subjects compared with a control condition without music or caffeine (CON).

Variable (Units)	CON	Mus + PLA	Mus + CAF	*p*	F
PP (W)	888.08 ± 133.20	943.95 ± 139.25 *	1017.39 ± 155.23 *** ^#^	<0.001	14.38
MP (W)	621.11 ± 75.55	657.65 ± 64.55 ***	665.41 ± 66.54 ***	<0.001	15.44
TW (J)	17,822.43 ± 2232.28	18,871.13 ± 1828.08 ***	19,160.08 ± 1960.68 ***	<0.001	13.36
FI (%)	61.39 ± 10.57	63.12 ± 8.69	64.70 ± 8.10	0.44	--
RPE (arbitrary units)	17.30 ± 1.29	17.52 ± 0.85	17.74 ± 0.81	0.37	--
HR (bpm)	184.35 ± 8.09	185.04 ± 8.46	185.86 ± 8.69	0.278	1.27

Note: Data are presented as means ± SDs. PP = peak power; MP = mean power; TW = total work; FI = fatigue index; RPE = rating of perceived exertion; HR = heart rate; CON = control; Mus = music; PLA = placebo; CAF = caffeine. * = *p* ≤ 0.05 for Mus + CAF or Mus + PLA compared with CON; *** = *p* < 0.001 for Mus + CAF or Mus + PLA compared with CON; ^#^ = *p* ≤ 0.05 for Mus + CAF compared with Mus + PLA.

**Table 3:** Effects of self-selected music combined with placebo (Mus + PLA) or with caffeine supplementation (Mus + CAF) on the Feeling Scale before and after the Wingate anaerobic test (WAnT) in physically active subjects compared with a control condition without music or caffeine (CON).

Variable (Units)	CON	Mus + PLA	Mus + CAF	*p*	F
FS–before (arbitrary units)	0.96 ± 1.02	2.21 ± 1.28 *	2.74 ± 1.25 ***	<0.001	--
FS–after (arbitrary units)	−2.09 ± 1.04	−2.52 ± 0.90	−2.39 ± 1.27	0.67	--

Note: The data are expressed as means ± SDs. FS = Feeling Scale; CON = control; Mus = music; PLA = placebo; CAF = caffeine. * = *p* ≤ 0.05 for Mus + CAF or Mus + PLA compared with CON; *** = *p* < 0.001 for Mus + CAF or Mus + PLA compared with CON.

**Table 4:** Ratings of perceived fitness following the Wingate anaerobic test, as well as the prevalence of side effects immediately and 24 h post-test under three conditions: self-selected music combined with placebo (Mus + PLA), self-selected music combined with caffeine supplementation (Mus + CAF), and a control condition without music or caffeine (CON).

Items (Units)	CON		Mus + PLA		Mus + CAF		*p* for After	*p* for 24 h After
	Just After	24 h After	Just After	24 h After	Just After	24 h After		
Power (arbitrary units)	5.09 ± 2.30	—	5.83 ± 1.30	—	6.43 ± 1.73 *	—	0.036	—
Endurance (arbitrary units)	4.70 ± 2.31	—	5.17 ± 1.70	—	5.52 ± 1.90	—	0.661	—
Fatigue (arbitrary units)	6.30 ± 1.99	—	6.70 ± 2.01	—	6.57 ± 1.93	—	0.615	—
Abdominal/gut discomfort (%)	4%	4%	13%	9%	17%	13%	0.882	0.607
Muscle soreness (%)	57%	65%	52%	70%	70%	78%	0.338	0.584
Increased urine output (%)	0%	0%	0%	9%	9%	17%	0.135	0.091
Headache (%)	0%	17%	4%	22%	0%	22%	0.368	0.882
Anxiety or nervousness (%)	0%	4%	0%	13%	9%	17%	0.135	0.311
Insomnia (%)	—	8%	—	13%	—	22%	—	0.459

Note: The data are expressed as means ± SDs for continuous variables and as percentages (%) for binary categorical variables related to side effects. CON = control; Mus = music; PLA = placebo; CAF = caffeine. * = *p* ≤ 0.05 for Mus + CAF or Mus + PLA compared with CON.

## Data Availability

Data supporting the findings of this study can be requested from the corresponding author (C.L.) upon reasonable justification. The data are not publicly available due to privacy concerns related to the inclusion of sensitive personal information.
